# Comparison of a Supraglottic Airway Device (v-gel^®^) with Blind Orotracheal Intubation in Rabbits

**DOI:** 10.3389/fvets.2017.00049

**Published:** 2017-04-10

**Authors:** Sarah Engbers, Amy Larkin, Nicolas Rousset, Melanie Prebble, Mahesh Jonnalagadda, Cameron G. Knight, Daniel S. J. Pang

**Affiliations:** ^1^Cochrane Veterinary Care Clinic, Cochrane, AB, Canada; ^2^Western College of Veterinary Medicine, University of Saskatchewan, Saskatoon, SK, Canada; ^3^Western Veterinary Specialist and Emergency Centre, Calgary, AB, Canada; ^4^University of Cincinatti, Cincinatti, OH, USA; ^5^University of Calgary Faculty of Veterinary Medicine (UCVM), Calgary, AB, Canada; ^6^Faculté de Médecine Vétérinaire, Université de Montréal, Saint-Hyacinthe, QC, Canada; ^7^Groupe de recherche en pharmacologie animale du Québec (GREPAQ), Université de Montréal, Saint-Hyacinthe, QC, Canada

**Keywords:** rabbit anesthesia, endotracheal intubation, secure airway, steroid anesthetic, alpha 2 agonists

## Abstract

**Introduction:**

Achieving a secure airway in rabbits is generally considered more difficult than in cats or dogs. Their relatively large tongue, small oropharyngeal cavity and glottis limit direct visualization. A rabbit-specific supraglottic airway device (SGAD) may offer benefits over blind orotracheal intubation.

**Animals and methods:**

Fifteen adult New Zealand white rabbits were randomized to SGAD or orotracheal intubation (ETT). All animals were sedated with dexmedetomidine (0.1 mg kg^−1^ IM) and midazolam (0.5 mg kg^−1^ IM), followed by induction with alfaxalone (0.3 mg kg^−1^ IV). Two CT scans of the head and neck were performed, following sedation and SGAD/ETT placement. The following were recorded: time to successful device insertion, smallest cross-sectional airway area, airway sealing pressure, and histological score of tracheal tissue. Data were analyzed with a Mann–Whitney test.

**Results:**

Two rabbits were excluded following failed ETT. Body masses were similar [ETT; *n* = 6, 2.6 (2.3–4.5) kg, SGAD; *n* = 7, 2.7 (2.4–5.0) kg]. SGAD placement was significantly faster [33 (14–38) s] than ETT [59 (29–171) s]. Cross-sectional area (CSA) was significantly reduced from baseline [12.2 (6.9–3.4) mm^2^] but similar between groups [SGAD; 2.7 (2.0–12.3) mm^2^, ETT; 3.8 (2.3–6.6) mm^2^]. In the SGAD group, the device tip migrated into the laryngeal vestibule in 6/7 rabbits, reducing the CSA. ETT airway seals were higher [15 (10–20) cmH_2_O], but not significant [SGAD; 5 (5–20) cmH_2_O, *p* = 0.06]. ETT resulted in significantly more mucosal damage [histological score 3.3 (1.0–5.0)], SGAD; 0.67 (0.33–3.67).

**Conclusion:**

The SGAD studied was faster to place and caused less damage than orotracheal intubation, but resulted in a similar CSA.

## Introduction

General anesthesia and sedation in rabbits carries a relatively high risk, with an overall incidence of death of 1.39% reported, approximately 6–8 times higher than that in cats and dogs (1). A range of factors potentially contribute to this increased risk, including difficulty achieving a secure airway, the propensity for hypoventilation during anesthesia, and underlying respiratory disease (1–5).

Orotracheal intubation provides a secure airway by facilitating the flow of gas during spontaneous ventilation, allowing intermittent positive pressure ventilation (IPPV), preventing aspiration of foreign material into the respiratory tract, and limiting workplace pollution with waste anesthetic gas. Achieving atraumatic, timely orotracheal intubation is difficult without training and practice and is hindered by the oropharyngeal anatomy of rabbits, with their relatively large tongues, narrow oropharyngeal cavity, and small glottis (5–11). Rabbits may be more susceptible to glottic or tracheal injury from endotracheal tubes because of a highly vascularized mucosa and submucosa compared to other species (7).

As a result of difficulties achieving orotracheal intubation, it is not commonly performed and anesthesia may be induced and maintained with a facemask; however, this commonly leads to inadequate ventilation (1, 12, 13). Supraglottic airway devices (SGADs) designed for humans have been successfully used during general anesthesia in rabbits and dogs and have the following advantages over orotracheal intubation with an endotracheal tube (ETT): rapid insertion by experienced and inexperienced operators alike, with a reduced anesthetic induction agent requirement, and reduced risk of airway trauma and cardiovascular stimulation during insertion (7, 8, 12, 14–16). However, lingual cyanosis, gastric tympany, and an incomplete airway seal have been reported with the use of human SGAD in rabbits (8, 12, 17). Recently, a rabbit-specific device (v-gel, Docsinnovent Ltd., London, UK) has been developed, which may help reduce these complications (6, 18).

The aim of this study was to assess the performance of this rabbit-specific SGAD against blind orotracheal intubation with an ETT. Primary outcome measures included time to successful device insertion, cross-sectional airway area, histological score, and airway seal. Compared with ETT, we hypothesized that the SGAD would be faster to place and maintain a larger cross-sectional airway area with less airway damage but create a less-effective airway seal.

## Animals and Methods

### Animals

In a prospective, randomised study design, adult New Zealand white rabbits were obtained as surplus stock from the Animal Resource Center of the University of Calgary and a commercial supplier (Charles River Laboratories, Québec city, QC, Canada, *n* = 12 males, *n* = 3 females). Rabbits from the commercial supplier had a minimum of 1 week to acclimatize before experimentation. Rabbits were either group or individually housed (depending on location) in an environmentally controlled unit [12 h light/12 h dark cycle (lights on at 0730), target temperature between 16 and 22°C, target humidity between 40 and 50%]. All rabbits were provided with *ad libitum* pellets, hay, and water with a small amount of fruit and vegetables. Experiments were performed between 1700 and 2000 hours.

Animals were block randomized to one of the two treatment groups for managing airway and each device type was placed by a single investigator: ETT (Daniel S. J. Pang) SGAD (Sarah Engbers). These investigators both completed an online training program provided by the manufacturer and practical experience was gained with two rabbits scheduled for euthanasia. Investigators could not be blinded to group assignment, with the exception of the veterinary pathologist (Cameron Knight), who performed necropsies off-site. Animals were fasted for approximately 2 h before the experiment. Physical examinations (cardiothoracic auscultation, examination of musculoskeletal system, and integument) were performed immediately before each experiment. Exclusion criteria included any indication of systemic illness resulting in assigning an American Society of Anesthesiologists physical classification status >2.

### Sedation

Animals were sedated with dexmedetomidine (0.1 mg kg^−1^, Dex-domitor, Zoetis, Kirkland, QC, Canada) and midazolam (0.5 mg kg^−1^, Midazolam Sandoz, Sandoz Canada, Boucherville, QC, Canada) delivered intramuscularly as a single injection in the epaxial musculature. Ten minutes later, animals were placed in sternal recumbency on the CT scan table, provided oxygen by facemask (1.5 L min^−1^) and cannulas (22 gauge 1″, BD Insyte, Becton Dickinson Infusion therapy systems Inc., Sandy, UT, USA) placed in the auricular vein (IV) and artery. A baseline CT scan (Toshiba Aquilon 8 slice, Markham, ON, Canada) was performed under sedation following instrumentation, followed by a second scan after ETT or SGAD placement. Scans were performed by a board-certified radiologist (Nicolas Rousset), with the scan margins ranging from the rostral margin of the nose to the thoracic inlet.

### Induction of Anesthesia and Device Insertion

Following the baseline CT scan, invasive systemic arterial blood pressure (BP), ECG, and saturation of arterial hemoglobin with oxygen (pulse oximetry probe on the prepuce or vulva) monitoring began (LifeWindow LW6000V, Digicare Animal Health, Boynton Beach, FL, USA). General anesthesia was induced with alfaxalone (0.3 mg kg^−1^ IV, Alfaxan, Jurox, Rutherford, NSW, Australia). The SGAD size was chosen based on body mass as per the manufacturer’s recommendations. Where an animal fell between sizes, the larger SGAD was selected. An insertion attempt began 30 s after completing alfaxalone injection. Each animal in the SGAD group was positioned in sternal recumbency with its tongue exteriorized and the lubricated SGAD (lubricating spray, Docsinnovent Ltd., London, UK) inserted into the oropharynx until either resistance was encountered, or the incisors were within 1–2 cm of the device’s fixation tabs. Acceptable placement was confirmed with a mainstream capnograph attached to the SGAD (Masimo EMMA Capnograph with pediatric sample chamber, Masimo, Danderyd, Sweden) and visualization of six rectangular waveforms. Criteria for rescue intubation with ETT for the SGAD group were defined as: lingual cyanosis that did not resolve with repositioning the SGAD or tongue, or three unsuccessful insertion attempts (no capnograph trace observed).

Orotracheal intubations were performed using a blind technique (19). Endotracheal tube size (2–3.5 mm ID, Sheridan/CF, Teleflex Medical Canada Inc., Markham, ON, Canada) was selected based on investigator experience. Each animal was positioned in sternal recumbency with the head and neck hyperextended. A single dose of lidocaine (12 mg lidocaine per metered dose, Lidodan, Odan Laboratories Ltd., Montreal, QC, Canada) was sprayed into the caudal oropharyngeal area 30 s before attempting ETT. Confirmation of ETT was with a mainstream capnograph, as described for SGAD placement. For both SGAD and ETT, the number of insertion attempts and total time for insertion were recorded. Insertion time was from the moment the SGAD or endotracheal tube passed the incisors until the first of six capnograph waveforms was observed. If capnograph waveforms were not observed following SGAD placement, adjustments were made by repositioning slightly rostrally or caudally. The number of attempts during intubation with each device was recorded, with an “attempt” representing the withdrawal of the device out of the mouth before re-inserting or changing to a different size. Airway devices were secured behind the ears with a bandage tie.

### Monitoring

Baseline systemic arterial BPs were recorded immediately before induction of general anesthesia. Beginning immediately after the insertion of the airway device, physiologic data (BP, heart, and respiratory rates, SpO_2_) were collected and recorded every 30 s for 5 min, and at 15 min post-insertion. Additionally, the highest systemic systolic arterial BP observed during device insertion was recorded (from video-recording of the physiologic monitor). At 10 min, the second CT scan was performed. At 15 min, the concentration of isoflurane measured from a sampling line placed approximately 5 cm from the mouth was recorded, and the airway seal was tested by closing the adjustable pressure limiting valve on the anesthetic breathing system and squeezing the reservoir bag. Peak inspiratory pressures were increased in 5 cmH_2_O increments, between 5 and 20 cmH_2_O, until a leak was heard (or 20 cmH_2_O achieved). This leak test was done by the same investigators (Sarah Engbers and Daniel S. J. Pang) for all animals. Occurrences of lingual cyanosis or airway obstruction (audible wheeze during respiratory cycle or irregular capnograph trace) were recorded. In the last nine animals (SGAD *n* = 4, ETT *n* = 5), an arterial blood sample was collected from the intra-arterial cannula 30 min after device placement and analyzed for blood gases and electrolytes (CG8+ cartridges, iSTAT Portable Clinical Analyzer, Heska Corporation, Fort Collins, CO, USA). General anesthesia was maintained for 60 min (from time of induction) with isoflurane (1% vaporizer setting) carried in oxygen (1 L min^−1^) through a non-rebreathing system (coaxial Bain). All animals were euthanized with an overdose of IV sodium pentobarbital while anesthetized at the end of the experiment.

### CT Image Reconstruction and Measurements

CT scans were reconstructed using a multi-planar reconstruction tool on OsiriX software (v 7.0.1, Pixmeo, Geneva, Switzerland).

**Figure 1:**
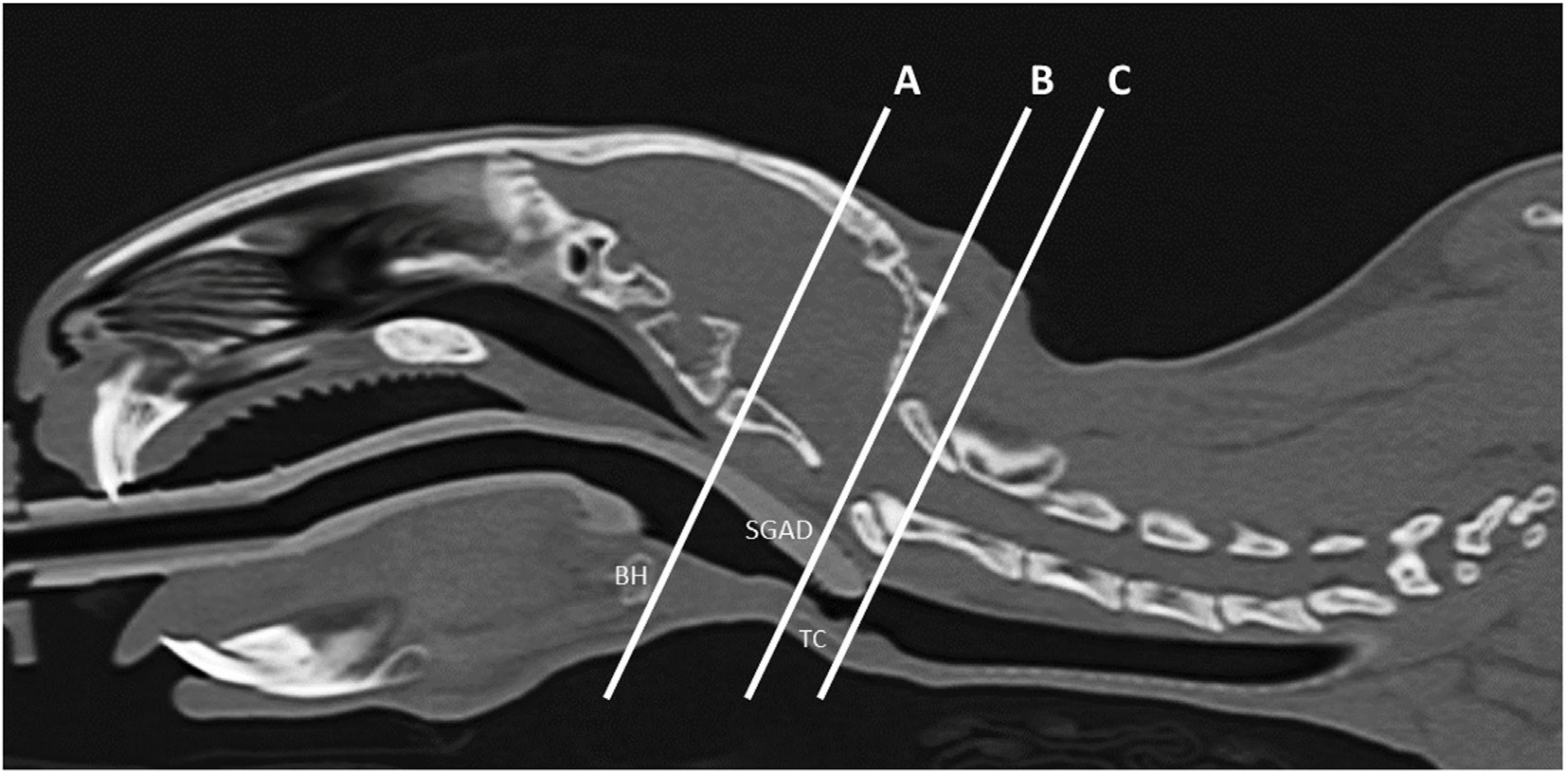
**Representative midline sagittal image showing the inserted supraglottic airway device (SGAD)**. The basihyoid bone (BH), thyroid cartilage (TC), and SGAD tip are labeled. The distance between the caudal edge of BH and rostral edge of TC is the distance A–B and was used to assess anatomical variation between rabbits. Insertion depth of the SGAD was measured by the distance A–C, the distance between the tip of the SGAD and the caudal edge of the BH.

**Figure 2:**
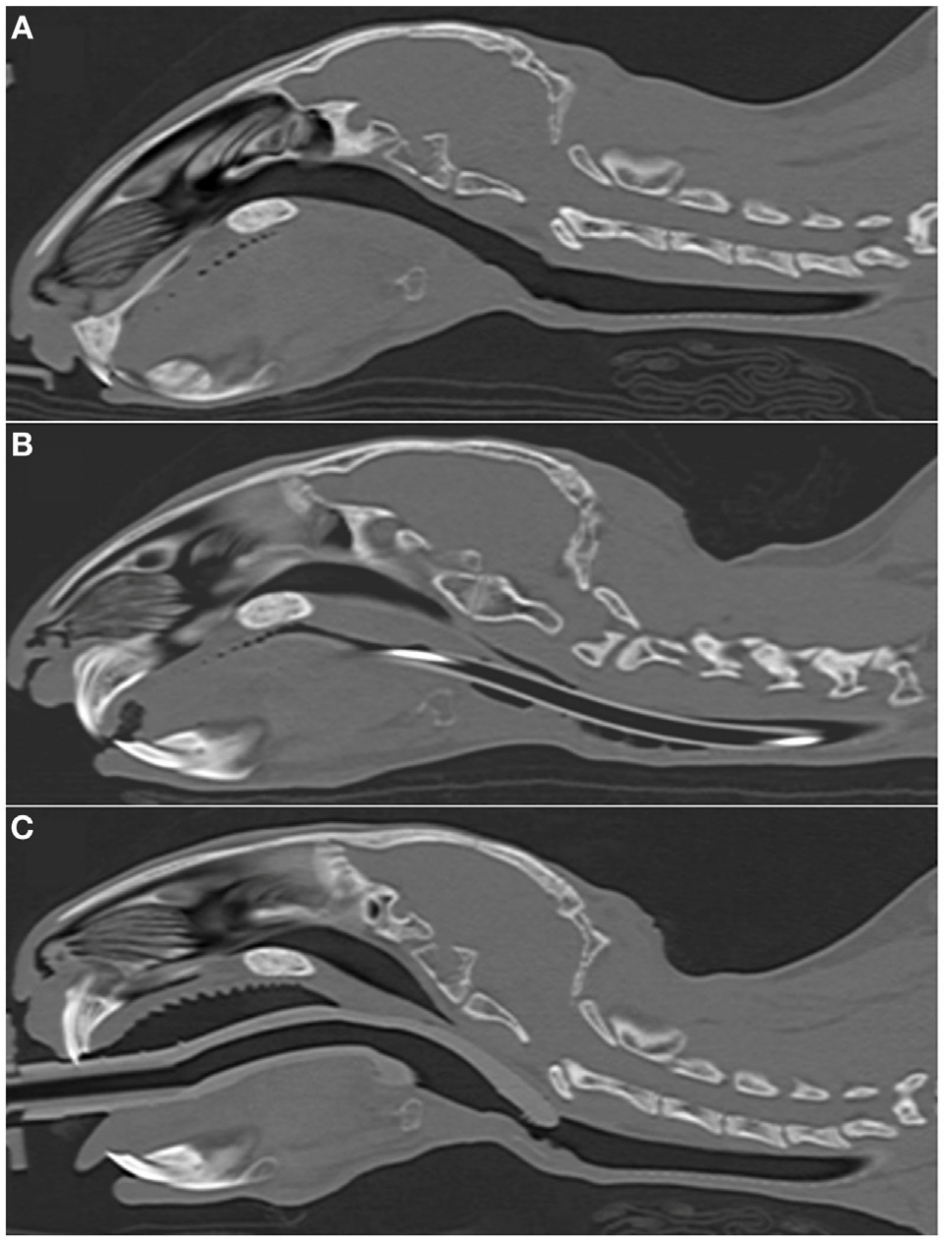
**Representative sagittal CT images of rabbits’ heads**. **(A)** 2.7 kg rabbit before airway device placement. **(B)** 2.7 kg rabbit with an endotracheal tube (2.5 mm ID) inserted into the trachea. **(C)** 2.7 kg rabbit [same animal as panel **(A)**] with a supraglottic airway device (SGAD) (SGAD, size R3) inserted. In panel **(B)**, the endotracheal tube is visible passing through the caudal oropharynx, larynx and trachea. Rostral to this, it lies outside the plane of view. In panel **(C)**, the tip of the SGAD enters the larynx, reducing the cross-sectional area (Figure 4).

**Figure 3:**
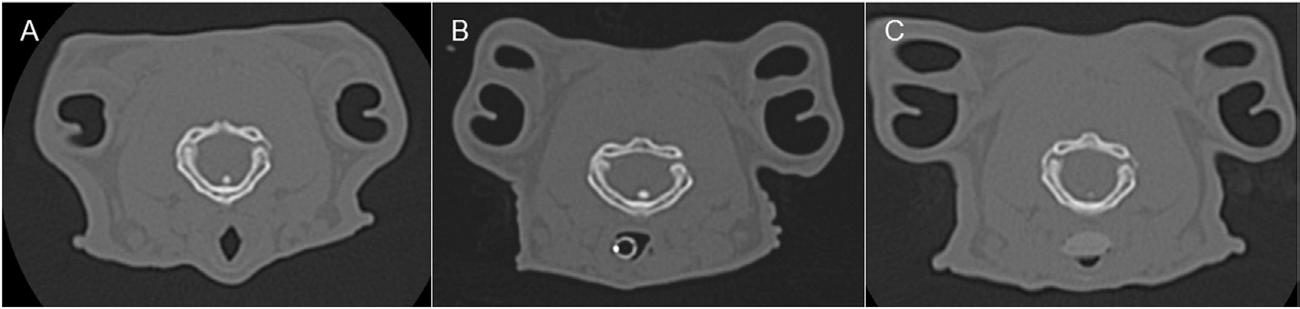
**Representative transverse CT images of the rabbits depicted in Figure 2, where (A) is before airway device placement, (B) is with the endotracheal tube inserted, and (C) is with the supraglottic airway device (SGAD) *in situ***. The image in **(C)** shows the narrowest cross-sectional area of the airway, including the SGAD tip. Panels **(A,B)** represent the same anatomical level as **(C)**.

All measurements were taken three times by a board-certified veterinary radiologist (Nicolas Rousset) and averaged for analysis. The midline sagittal view of each SGAD rabbit post-device placement was used to measure the following distances: (1) distance between the caudal edge of the basihyoid bone (BH) to the rostral edge of the thyroid cartilage (TC) (A–B, Figure 1), (2) the distance from the caudal edge of the BH to the tip of the SGAD (A–C, Figure 1), and (3) the distance between the SGAD tip and the rostral edge of the TC (B–C, Figure 1). Confirmation of whether the SGAD tip entered the upper esophageal sphincter was recorded in each instance, as well as the anatomical location of the SGAD tip. Transverse images from before and after device placement were compared to determine the narrowest region of the upper airway. In the SGAD group, the site of the narrowest section was also measured relative to the SGAD tip in order to determine any contribution from the device to airway narrowing. The cross-sectional area (CSA) of the narrowest point was measured. For the ETT group, the luminal CSA was measured.

### Necropsies

Necropsies were performed on all rabbits within 2 h of euthanasia by a board-certified veterinary pathologist (Cameron Knight) according to a standardized method (20). The tongue, pharynx, larynx, and trachea were collected and fixed in 10% neutral-buffered formalin. Tissues were prepared routinely for histologic evaluation and stained with hematoxylin and eosin. For each rabbit, three to four representative transverse sections from each of the following locations were examined histologically: (1) larynx at the level of the vocal folds, (2) trachea immediately caudal to the larynx, and (3) trachea 1 cm caudal to the larynx. The mucosal and submucosal layers in each section were evaluated according to a scoring system modified from a previously published study (7). The modification involved removing scoring criteria that reflected chronic injury from repeated endotracheal intubation over several weeks, which were not applicable in the current study. The scoring criteria used in this study are listed in Table 1. Scores for sections from the same region were averaged and a total score (/6) was calculated by summing the mucosal and submucosal scores.

**Table 1:** **Histological scale used to score damage to mucosal and submucosal layers of the larynx and cranial third of the trachea**.

Mucosa	Submucosa	Score
Normal	Normal	0
Mild focal erosion with little or no leukocytic infiltration	Minimal to mild locally extensive congestion	1
(Multi)focal erosion or ulceration with edema of lamina propria, moderate mixed leukocytic infiltration, and hemorrhage	Moderate diffuse congestion and mild perivascular edema	2
Extensive erosion or ulceration with marked mixed leukocytic infiltration, cellular debris, hemorrhage, and possibly surface exudate	Moderate diffuse congestion and edema with or without hemorrhage, and leukocyte infiltration associated with overlying ulcer or erosion	3

*The scale has been modified from that of Phaneuf et al. (7)*.

### Statistics

Commercial software was used for statistical analyses (Prism v 7.0a, GraphPad Software, La Jolla, CA, USA). Data were tested for normal distribution with a D’Agostino and Pearson normality test. Non-parametric analyses were used if data were not normally distributed or sample sizes were too small for the normality test. Mann–Whitney tests were used to make the following comparisons between groups: body mass, anatomical distances, cross-sectional airway areas, device insertion time, airway seal, percentage changes in systolic BP during device insertion, arterial blood sample parameters, and histological scores. A Wilcoxon matched pairs test was used to compare changes in systolic arterial BP over time. Two-way ANOVA was applied to systemic arterial BPs, heart rate, respiratory rate, and expired CO_2_ data, followed by a Bonferroni *post hoc* test if main effects were significant. A *p* value < 0.05 was considered significant. Data are presented as mean ± SD and median (range). 95% confidence intervals (95% CI) for mean or median differences are provided as available.

## Results

### Animals

Two rabbits were excluded from the ETT group, one due to failure to achieve orotracheal intubation and the other due to prolonged intubation resulting from fecal matter in the oropharynx (21). CT scans were not performed in two rabbits (both from the ETT group) due to unavailability of the CT scanner. In these cases, the anesthetic duration was matched to the full procedure and other data were collected as planned. All rabbits in the SGAD group had a device placed. Final group sizes were: ETT, *n* = 6; SGAD, *n* = 7. Rabbits ranged in age from 3 to 12 months. There were no significant differences in body mass between groups: ETT; 2.6 (2.3–4.5) kg, SGAD; 2.7 (2.4–5.0) kg (*p* = 0.38, 95% CI −2.3 to 1.8). All rabbits appeared healthy on physical examination and were assigned an American Society of Anesthesiologists physical classification status of 1. The endotracheal tubes used in the ETT group ranged from 2 to 3.5 mm ID: 2 mm, *n* = 2; 2.5 mm, *n* = 1; 3 mm, *n* = 1; 3.5 mm, *n* = 2. Three sizes of SGAD were used: R3, *n* = 4; R4, *n* = 1; and R5, *n* = 2.

### CT Imaging

Airway anatomy was similar between groups. The distance between the caudal edge of the BH and the rostral edge of the TC did not differ significantly (*p* = 0.16, 95% CI −6.7 to 1.3): SGAD, 16.2 (13.2–20.3) mm; ETT, 14.5 (12.7–15.8) mm (distance A–B, Figure 1).

**Figure 4:**
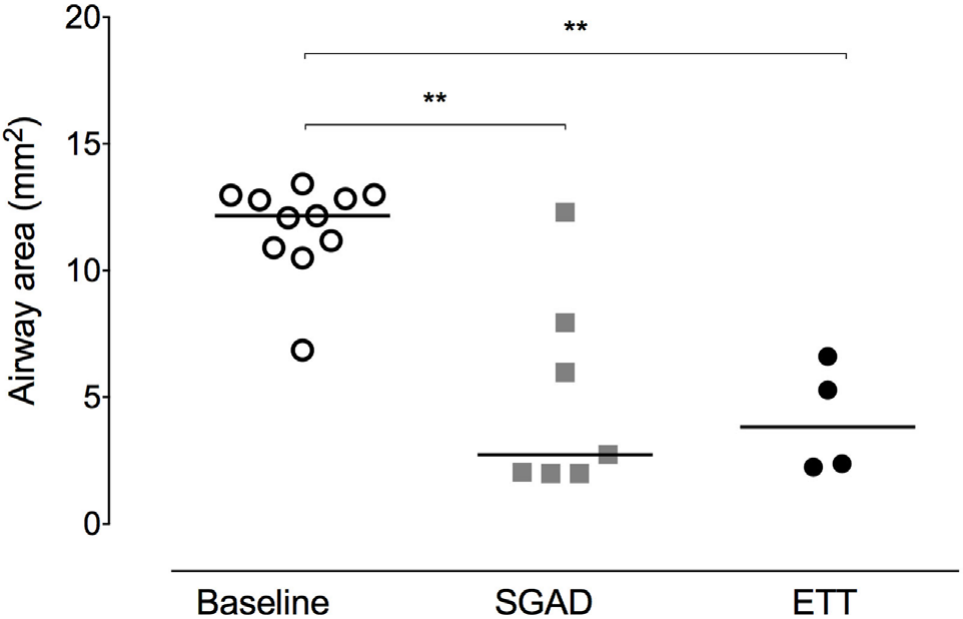
**Scatter plot of cross-sectional areas (CSA) at the narrowest point of the upper airway before (baseline) and after supraglottic airway device (SGAD) or endotracheal tube (ETT) placement**. CSA of pre-intubation versus SGAD, *p = 0.003. CSA of pre-intubation versus ETT, *p = 0.001. Horizontal line is median.

In most cases (6/7 rabbits), placement of the SGAD resulted in the tip of the device deviating ventrally to be positioned within the laryngeal vestibule (*n* = 6, Figures 2 and 3). This resulted in a reduction in the CSA of the airway. The CSA before device placement was smallest within the nasopharynx in nine rabbits and within the larynx in two rabbits, with no significant difference between groups (*p* = 0.78, 95% CI −2.3 to 3.7 mm^2^) so that baseline CSA was pooled. The CSA of the narrowest point decreased significantly between the baseline CT scan [CSA, 12.2 (6.9–13.4) mm^2^] and placement of the SGAD [CSA, 2.7 (2.0–12.3) mm^2^, *p* = 0.003, 95% CI 4.1 to 10.3] and ETT [CSA, 3.8 (2.3–6.6) mm^2^, *p* = 0.002, 95% CI 4.6–10.6, Figure 4]. There was no significant difference in CSA between ETT and SGAD (*p* = 0.93, 95% CI −5.7 to 3.9). The site of the smallest CSA in the SGAD group was located within the device tip [4.2 mm (range 3.2–5.2) from the tip extremity] at an average of 5.3 mm (range 2.9–7.9) caudal to the rostral edge of the TC, so that the smallest CSA was formed by the SGAD dorsally and the rabbit’s airway ventrally (Data Sheet S1 in Supplementary Material). In the ETT group, a uniform CSA was created by the endotracheal tube.

### Device Placement

**Figure 5:**
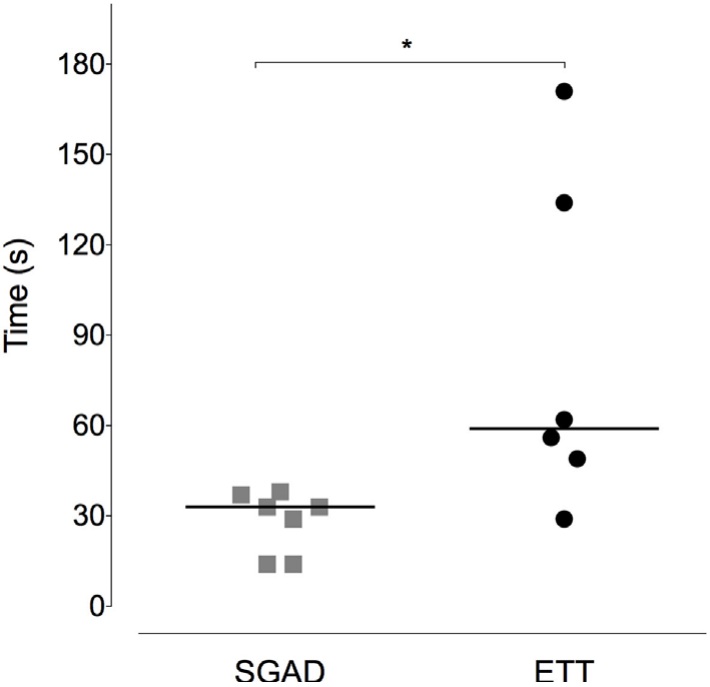
**Scatter plot of time to insert the supraglottic airway device (SGAD) and endotracheal tube (ETT)**. *p = 0.02. Horizontal line is median.

**Figure 6:**
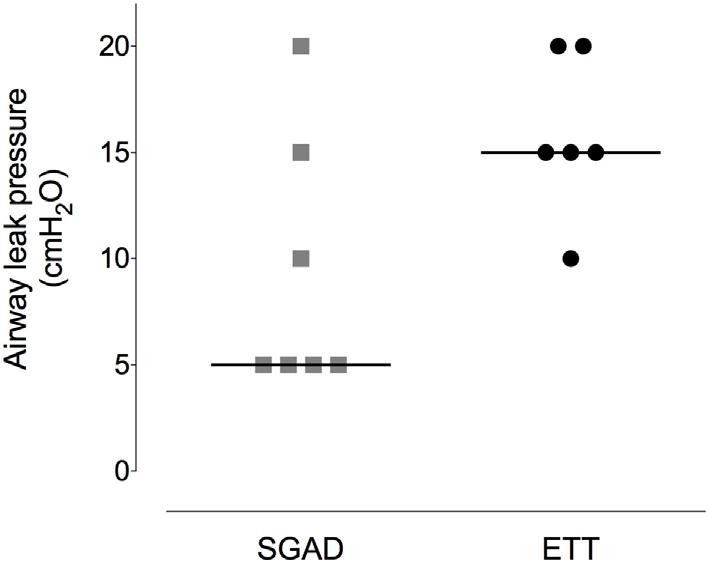
**Scatter plot of the pressure at which a leak was detected when testing the airway seal in the supraglottic airway device (SGAD) and endotracheal tube (ETT) groups**. *p = 0.02. Horizontal line is median.

Device insertion time was shorter and more consistent (narrower range) for the SGAD [33 (14–38) s] than ETT [59 (29–171) s, *p* = 0.02, 95% CI 12–133, Figure 5]. The number of attempted device placements did not differ between groups (SGAD: 1 attempt, *n* = 4; 2 attempts, *n* = 3. ETT: 1 attempt, *n* = 4; 2 attempts, *n* = 2. *p* > 0.99, 95% CI −1 to 1). The median airway seal was higher in the ETT group [15 (10–20) cmH_2_O], but there was no statistical difference compared to the SGAD group [5 (5–20) cmH_2_O, *p* = 0.06, 95% CI 0–15, Figure 6]. A single animal in the SGAD group had an airway seal of 20 cmH_2_O. In this animal, loss of the capnograph trace indicated respiratory obstruction, so the SGAD was retracted until the trace returned and the airway seal test performed after repositioning. The median SGAD insertion depth was 20.7 (11.1–26.5) mm (distance A–C, Figure 1), and the rabbit in which the SGAD was adjusted had the shortest insertion distance (11.1 mm), so that the device tip did not enter the larynx. Isoflurane was not detected, nor lingual cyanosis observed, in any rabbit. Lingual cyanosis was not observed in any rabbit of either treatment group.

### Histological Evaluation

**Figure 7:**
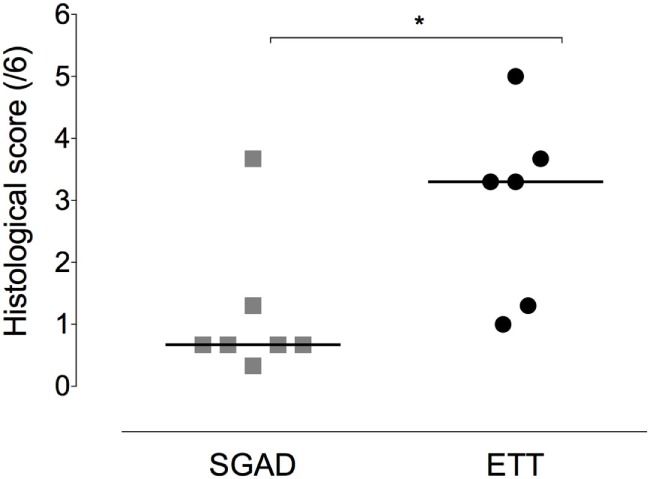
**Scatter plot of histopathology scores for the supraglottic airway device (SGAD) and endotracheal intubation (ETT) groups**. *p = 0.03. Horizontal line is median. Score range is 0–6, with increasing scores reflecting increasing submucosal and mucosal damage.

The ETT group was found to have significantly more damage to tracheal mucosa and submucosa [3.3 (1.0–5.0)] than the SGAD group [0.67 (0.33–3.67), *p* = 0.03, 95% CI 0–3.3, Figure 7].

### Physiologic Variables

**Figure 8:**
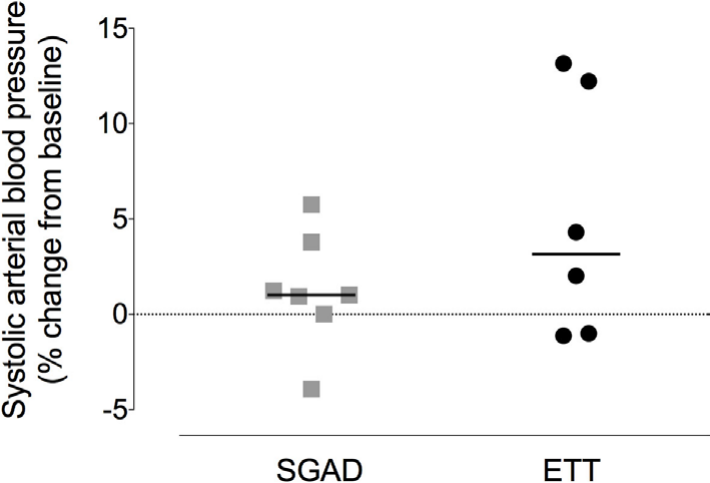
**Scatter plot of percentage change in systolic arterial blood pressure for the supraglottic airway device (SGAD) and endotracheal intubation (ETT) groups during placement of each device**. *p* = 0.2. Horizontal line is median.

There was a systematic, albeit non-significant, difference in systemic arterial BP between groups, with higher BPs observed in the SGAD group (systolic, *p* = 0.45; diastolic, *p* = 0.11; mean, *p* = 0.17; Data Sheet S2 in Supplementary Material). The percentage change in peak systolic arterial BP observed during placement did not differ between each group and baseline (SGAD, *p* = 0.3, 95% CI −5.8 to 3.9; ETT, *p* = 0.16, 95% CI −13.2 to 1.1). Between groups, ETT [3.2 (−1.1 to 13.2)%] showed a wider range of percentage change in peak systolic BP than SGAD [1.0 (−3.9 to 5.8)%] though the median difference was not significant (*p* = 0.45, 95% CI −11.9 to 2.1, Figure 8). There were no significant main effects between treatment groups for heart rate (*p* = 0.32), respiration rate (*p* = 0.22), and expired CO_2_ (*p* = 0.74), though heart and respiratory rates tended to be higher in the SGAD group (Data Sheet S2 in Supplementary Material). One rabbit in each group had a single SpO_2_ reading <90% (SGAD, 89%; ETT, 88%; both were recorded immediately following device insertion).

### Blood Gases and Electrolytes

No significant differences in the following variables were observed: pH, PaO_2_, PaCO_2_, BE, HCO_3_, SaO_2_, Na, K, iCa, hema-tocrit, hemoglobin (Table S1 in Supplementary Material). Hypercapnea was present in both treatment groups: SGAD; 65.1 (54.0–75.1) mmHg, ETT; 51.8 (41.2–67.8) mmHg.

## Discussion

The findings of this study show that, in comparison with orotracheal intubation using a blind technique, the rabbit-specific SGAD studied is rapid to place and causes minimal trauma to the upper airway. As with orotracheal intubation, the SGAD can be used to successfully maintain general anesthesia in rabbits.

General anesthesia and sedation in rabbits carries an unacceptably high mortality rate compared with dogs and cats. A study of 8,209 rabbits identified respiratory and cardiovascular or respiratory causes (where the initial problem could not be discerned) as the most frequent causes (13 and 23%, respectively) (1). Underlying respiratory disease, respiratory depression during anesthesia and absence of a secure airway have been suggested as contributing factors (1–5).

Providing a secure airway and the ability to support ventilation pose a challenge to anesthetists inexperienced with rabbits and when blind orotracheal intubation is attempted. Achieving orotracheal intubation is difficult without training and practice (8, 22). Compared with dogs and cats, direct visualization without appropriate equipment is hindered by a relatively large tongue, narrow oropharyngeal cavity, and small glottis (5–11). When using a blind intubation technique, multiple intubation attempts, intubation times of several minutes, and the occasional failure are commonly reported (8, 14, 22). Our findings reflect this, with improved consistency in the time taken to secure an airway with the SGAD, in which the longest time was 38 s, compared with 171 s in the ETT group. This difference is magnified when the case of failed intubation is considered. This advantage of the SGAD is particularly important, as the time taken to achieve orotracheal intubation by novices can be substantially longer (240–360 s in one study) than the range reported here (8). Furthermore, successful orotracheal intubation may require multiple attempts, potentially contributing to postoperative morbidity and mortality. Though the number of attempts did not differ between SGAD and ETT, our results show that even one to two attempts with ETT resulted in measurable trauma to the tracheal tissues. Similar findings have been reported in horses and our findings are consistent with those of Phaneuf et al., where histologic changes were reported after a single intubation event (the number of attempts was not reported) using both cuffed and uncuffed endotracheal tubes (7, 23). Since animals were euthanized at the end of our study, the consequences of the histologic changes are unknown; however, morbidity and mortality have been reported following apparently uneventful blind orotracheal intubations (7, 24). One rabbit in the SGAD group had a higher histological score, of 3.67, than the rest of the group. The location of the damaged mucosa was on the dorsal wall of the airway, beginning 10 mm caudal to the basihyoid and extending approximately 40 mm caudally. The cause of this lesion is unclear as the SGAD tip did not extend this far caudally.

With regular use, it is possible that the time to place the SGAD may be reduced further, and recorded times in this study were increased because they included observing a waveform on the capnograph (18). Securing an airway in a reasonable time allows management of hypoventilation and control of PaCO_2_ and PaO_2_.

Hypoventilation is a common occurrence at induction and during general anesthesia in rabbits (2–4, 12). A frequent alternative to orotracheal intubation is the use of a facemask. This has several disadvantages, including lack of a secure airway, potential workplace pollution, and the inability to support ventilation. The use of a facemask does not allow IPPV to be performed and increases mechanical deadspace, both of which contribute to hypercapnea (in excess of 100 mmHg), respiratory acidosis, and increased risk of hypoxemia (12). When a SGAD is placed, as in this study, hypercapnea may still occur without IPPV (12). As IPPV was not instituted in this study, it was not possible to assess if the relatively low airway sealing pressures generated were sufficient; however, they did prevent measurable levels of isoflurane at a distance comparable to the head of an operator during dental procedures. Higher sealing pressures were identified in a feline version of the SGAD, with 7/15 animals maintaining a seal up to a peak inspiratory pressure of 16 cmH_2_O (25). A limitation of the present study was that repositioning of the SGAD to try and achieve a higher sealing pressure was not done.

The tip of the SGAD studied is designed to enter the upper esophagus during placement and act as a seal to ensure gas flow is directed in to the airways. Our results suggest that this does not occur. Explanations for this discrepancy may include placement technique, equipment, and anatomical differences. Both authors involved in device placement completed an online training course available on the manufacturer’s website as well as successfully performing several SGAD placements in two rabbits scheduled for euthanasia. During product development, the device designer suggested that a degree of experience is necessary for optimal placement in combination with capnograph trace evaluation (18). High fresh gas flow rates, large sampling chambers, and slow rise times can distort capnograph traces, particularly in smaller animals, though the gas flow rate used was comparable to that previously described in rabbits (1.5–2 L min^−1^), a small sample chamber (dead space volume of 1 mL) was used and the rise time of the capnograph is reported as <60 ms (for 10–90% step change). Optimization of SGAD placement by confirming clear breath sounds with a stethoscope placed on the larynx has also been suggested (12). Training with an experienced operator may have improved placement though the high incidence of SGAD tip deviation toward the larynx suggests that device material or shape were a contributing factor. SGAD size was selected based on manufacturer guidelines. All rabbits in this study were of the same breed and while it is unknown if this breed was included during device design, airway management with this SGAD has been successful in this breed in other studies (14, 15).

Laryngoscopy and endotracheal intubation are associated with a hemodynamic response (tachycardia and arterial hypertension) in humans, with associated morbidity and mortality from arrhythmias, intracranial hemorrhage, and elevated intracranial pressure (26–28). The use of SGAD in humans reduces this response and while our data are inconclusive, this deserves further study (29).

In conclusion, these results show the rabbit-specific SGAD studied to be consistently faster to place and to cause less trauma to laryngeal and tracheal tissue than a blind orotracheal intubation technique. The SGAD was capable of maintaining general anesthesia, though the presence of hypercapnea underlines the importance of the option to provide IPPV.

## Ethics Statement

Institutional ethics approval was provided by the Veterinary Science Animal Care Committee of the University of Calgary, which operates in accordance with the Canadian Council on Animal Care (VSACC AC14-0229).

## Author Contributions

Study design: SE, AL, MJ, NR, CK, and DP. Data collection: all authors. Data analysis and interpretation: SE, AL, NR, CK, and DP. Manuscript drafting and final approval: all authors.

## Conflict of Interest Statement

The authors declare that the research was conducted in the absence of any commercial or financial relationships that could be construed as a potential conflict of interest.

## Supplementary Material

Video S1 **3D image reconstruction of supraglottic airway device (v-gel) placement in an adult New Zealand white rabbit**.

## References

[1] Brodbelt DC Blissitt KJ Hammond RA Neath PJ Young LE Pfeiffer DU The risk of death: the confidential enquiry into perioperative small animal fatalities. Vet Anaesth Analg (2008) 35:365–73. 10.1111/j.1467-2995.2008.00397.x 18466167

[2] Zornow MH. Ventilatory, hemodynamic and sedative effects of the alpha 2 adrenergic agonist, dexmedetomidine. Neuropharmacology (1991) 30:1065–71. 10.1016/0028-3908(91)90135-X 1684646

[3] Navarrete-Calvo R Gómez-Villamandos RJ Morgaz J Manuel Domínguez J Fernández-Sarmiento A Muñoz-Rascón P Cardiorespiratory, anaesthetic and recovery effects of morphine combined with medetomidine and alfaxalone in rabbits. Vet Rec (2014) 174:95. 10.1136/vr.101293 24408312

[4] Huynh M Poumeyrol S Pignon C Le Teuff G Zilberstein L. Intramuscular administration of alfaxalone for sedation in rabbits. Vet Rec (2015) 176:255. 10.1136/vr.102522 25433053

[5] Flecknell P Anaesthesia and perioperative care. In: Meredith A Flecknell P, editors. BSAVA Manual of Rabbit Medicine and Surgery. Quedgeley, UK: British Small Animal Veterinary Association (2006). p. 154–65.

[6] Crotaz IR Initial feasibility investigation of the v-gel airway: an anatomically designed supraglottic airway device for use in companion animal veterinary anaesthesia. Vet Anaesth Analg (2010) 37:579–80. 10.1111/j.1467-2995.2010.00566.x 21040382

[7] Phaneuf LR Barker S Groleau MA Turner PV. Tracheal injury after endotracheal intubation and anesthesia in rabbits. J Am Assoc Lab Anim Sci (2006) 45:67–72. 17089996

[8] Smith JC Robertson LD Auhll A March TJ Derring C Bolon B. Endotracheal tubes versus laryngeal mask airways in rabbit inhalation anesthesia: ease of use and waste gas emissions. Contemp Top Lab Anim Sci (2004) 43:22–5. 15264765

[9] Abumandour MMA El-Bakary RMA Anatomic reference for morphological and scanning studies of the New Zealand white rabbits tongue (*Orycotolagus cuniculus*) and their lingual adaptation for feeding habits. J Morphol Sci (2013) 30:254–65.

[10] Johnson DH. Endoscopic intubation of exotic companion mammals. Vet Clin North Am Exot Anim Pract (2010) 13:273–89. 10.1016/j.cvex.2010.01.010 20381777

[11] Tran HS Puc MM Tran JL Del Rossi AJ Hewitt CW. A method of endoscopic endotracheal intubation in rabbits. Lab Anim (2001) 35:249–52. 10.1258/0023677011911705 11459409

[12] Bateman L Ludders JW Gleed RD Erb HN. Comparison between facemask and laryngeal mask airway in rabbits during isoflurane anesthesia. Vet Anaesth Analg (2005) 32:280–8. 10.1111/j.1467-2995.2005.00169.x 16135209

[13] Brodbelt D Perioperative mortality in small animal anaesthesia. Vet J (2009) 182:152–61. 10.1016/j.tvjl.2008.06.011 18658000

[14] Toman H Erbas M Sahin H Kiraz HA Uzun M Ovali MA. Comparison of the effects of various airway devices on hemodynamic response and QTc interval in rabbits under general anesthesia. J Clin Monit Comput (2015) 29:727–32. 10.1007/s10877-015-9659-x 25637244

[15] Uzun M Kiraz HA Ovali MA Sahin H Erbas M Toman H. The investigation of airway management capacity of v-gel and cobra-PLA in anaesthetised rabbits. Acta Cir Bras (2015) 30:80–6. 10.1590/S0102-86502015001000011 25627275

[16] Wiederstein I Auer U Moens Y. Laryngeal mask airway insertion requires less propofol than endotracheal intubation in dogs. Vet Anaesth Analg (2006) 33:201–6. 10.1111/j.1467-2995.2005.00254.x 16764583

[17] Kazakos GM Anagnostou T Savvas I Raptopoulos D Psalla D Kazakou IM. Use of the laryngeal mask airway in rabbits: placement and efficacy. Lab Anim (NY) (2007) 36:29–34. 10.1038/laban0407-29 17380146

[18] Crotaz IR An observational clinical study in cats and rabbits of an anatomically designed supraglottic airway device for use in companion animal veterinary anaesthesia. Vet Rec (2013) 172:606 10.1136/vr.100668 23677644

[19] Fick TE Schalm SW. A simple technique for endotracheal intubation in rabbits. Lab Anim (1987) 21:265–6. 10.1258/002367787781268837 3626474

[20] King JM Roth-Johnson L Dodd DC Newsom ME The Necropsy Book: A Guide for Veterinary Students, Residents, Clinicians, Pathologists, and Biological Researchers. Ithaca: The Internet-First University Press (2014).

[21] Engbers S Larkin A Jonnalagadda M Prebble M Rousset N Knight CG Difficult orotracheal intubation in a rabbit resulting from the presence of faecal pellets in the oropharynx. Vet Rec Case Rep (2016) 4:e000265 10.1136/vetreccr-2015-000265

[22] Grint NJ Murison PJ. A comparison of ketamine-midazolam and ketamine-medetomidine combinations for induction of anaesthesia in rabbits. Vet Anaesth Analg (2008) 35:113–21. 10.1111/j.1467-2995.2007.00362.x 18179655

[23] Heath RB Steffey EP Thurmon JC Wertz EM Meagher DM Hyyppa T Laryngotracheal lesions following routine orotracheal intubation in the horse. Equine Vet J (1989) 21:434–7. 10.1111/j.2042-3306.1989.tb02190.x 2591359

[24] Grint NJ Sayers IR Cecchi R Harley R Day MJ. Postanaesthetic tracheal strictures in three rabbits. Lab Anim (2006) 40:301–8. 10.1258/002367706777611415 16803648

[25] Prasse SA Schrack J Wenger S Mosing M. Clinical evaluation of the v-gel supraglottic airway device in comparison with a classical laryngeal mask and endotracheal intubation in cats during spontaneous and controlled mechanical ventilation. Vet Anaesth Analg (2016) 43:55–62. 10.1111/vaa.12261 25819338

[26] Khan FA Ullah H. Pharmacological agents for preventing morbidity associated with the haemodynamic response to tracheal intubation. Cochrane Database Syst Rev (2013) CD004087. 10.1002/14651858.CD004087.pub2 23824697 PMC11822245

[27] King BD Harris LC Greifenstein FE Elder JD Dripps RD Reflex circulatory responses to direct laryngoscopy and tracheal intubation performed during general anesthesia. Anesthesiology (1951) 12:556–66. 14868891 10.1097/00000542-195109000-00002

[28] Shribman AJ Smith G Achola KJ. Cardiovascular and catecholamine responses to laryngoscopy with and without tracheal intubation. Br J Anaesth (1987) 59:295–9. 10.1093/bja/59.3.295 3828177

[29] Ismail SA Bisher NA Kandil HW Mowafi HA Atawia HA. Intraocular pressure and haemodynamic responses to insertion of the i-gel, laryngeal mask airway or endotracheal tube. Eur J Anaesthesiol (2011) 28:443–8. 10.1097/EJA.0b013e328345a413 21455075

