# Supplementary material for: Comparison of a Supraglottic Airway Device (v-gel®) with Blind Orotracheal Intubation in Rabbits
Source: Front Vet Sci. 2017 Apr 10;4:49. doi: 10.3389/fvets.2017.00049 (PMC5385366; doi:10.3389/fvets.2017.00049)
Supplement: Supplementary file 2 [file Data_Sheet_1.pdf]

Parameter	SGAD<br>**Median (range)**	ETT<br>**Median (range)**	P value	95%CI
pH	7.36 (7.32 - 7.40)	7.38 (7.36 - 7.47)	0.69	-0.04 to 0.15
PaCO2 (mmHg)	65.1 (54.0 - 75.1)	51.8 (41.2 - 67.8)	0.20	-33.9 to 13.8
PaO2 (mmHg)	302.5 (207.0 - 428.0)	167.0 (117.0 - 305.0)	0.11	-311 to 98
BE (mmolL^{-1})	12 (9 - 15)	8.5 (-1 - 15)	0.34	-16 to 6
HCO3 (mmolL^{-1})	36.4 (34.8 - 41.0)	32.8 (24.3 - 40.0)	0.20	-16.7 to 5.2
Glucose mg/dL	366.5 (363.0 - 427.0)	367.5 (271.0 - 421.0)	0.69	-156 to 58
SaO2 (%)	100.0 (100.0 - 100.0)	99.5 (98.0 - 100.0)	0.43	-2 to 0
Na (mmolL^{-1})	143 (140 - 144)	144 (142 - 148)	0.49	-2 to 8
K (mmolL^{-1})	3.9 (3.6 - 4.0)	3.6 (3.2 - 3.8)	0.11	-0.8 to 0.2
iCa (mmolL^{-1})	1.7 (1.5 - 1.8)	1.6 (1.5 - 1.7)	0.31	-0.3 to 0.22
Hct (%PCV)	36 (35 - 38)	33 (29 - 38)	0.34	-9 to 3
Hb (g/dL)	12.2 (11.9 - 12.9)	11.2 (9.9 - 12.9)	0.34	-3 to 1

Supplementary Data S1: Blood gas and electrolyte data from arterial blood samples drawn 30 minutes after either endotracheal intubation (ETT) or placement of a supraglottic airway device (SGAD).
