# Supplementary material for: Comparison of a Supraglottic Airway Device (v-gel®) with Blind Orotracheal Intubation in Rabbits
Source: Front Vet Sci. 2017 Apr 10;4:49. doi: 10.3389/fvets.2017.00049 (PMC5385366; doi:10.3389/fvets.2017.00049)
Supplement: Supplementary file 3 [file Data_Sheet_2.pdf]

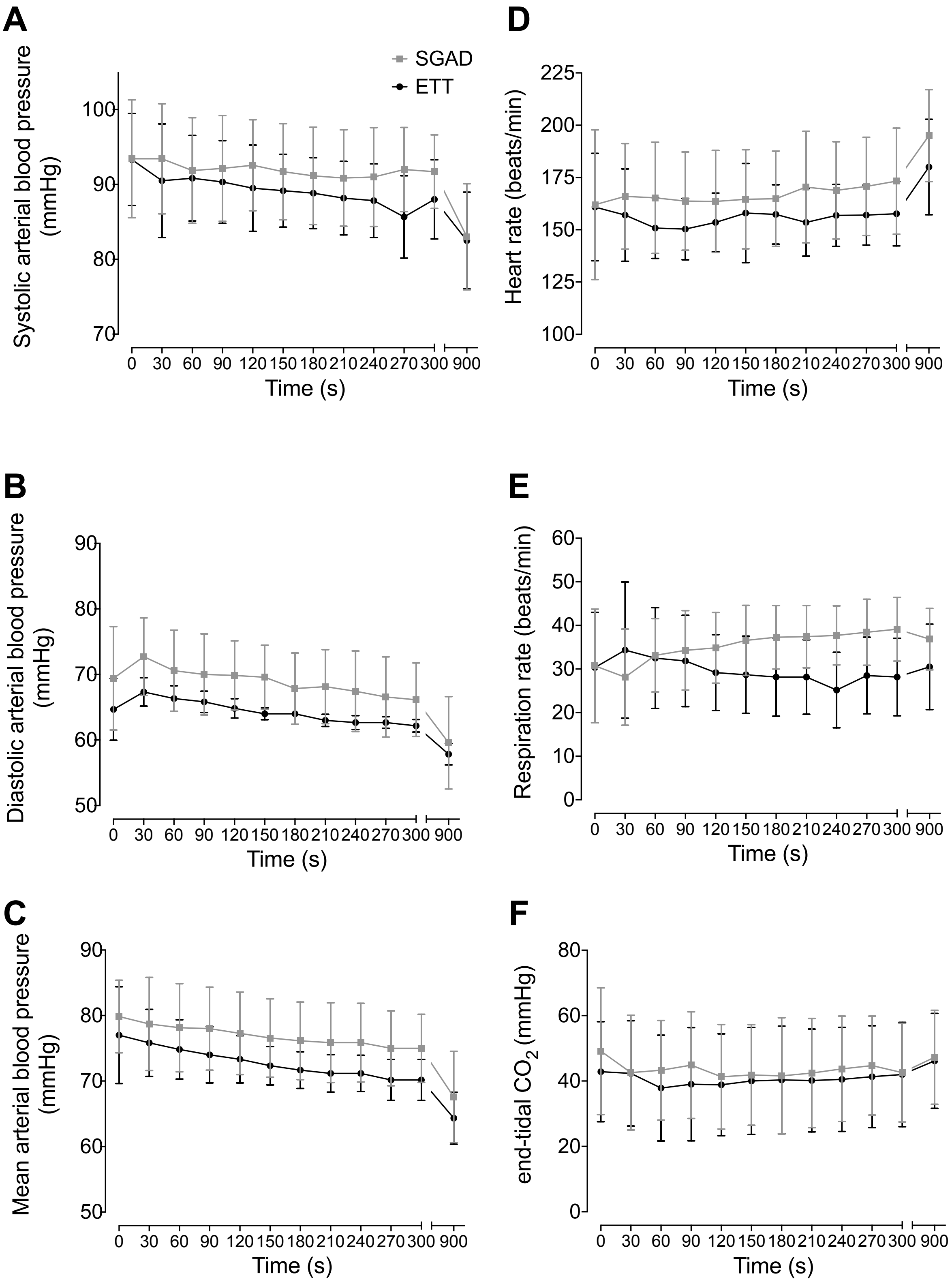

Supplementary Data S2: Cardiopulmonary data for the supraglottic airway device (SGAD, grey squares) and endotracheal intubation (ETT, black circles) groups. A: systolic arterial blood pressure (main effect, p = 0.45). B: diastolic arterial blood pressure (main effect, p = 0.11). C: mean arterial blood pressure (main effect, p = 0.17). D: heart rate (main effect, p = 0.32). E: respiration rate (main effect, p = 0.22). F: partial pressure of end-tidal carbon dioxide (main effect, p = 0.74). Data are mean ± SD.
